# Blunt bilateral diaphragmatic rupture—A right side can be easily missed

**DOI:** 10.1016/j.tcr.2015.10.006

**Published:** 2015-11-26

**Authors:** Maria Michailidou, Narong Kulvatunyou, Bellal Joseph, Lynn Gries, Randall S. Friese, Donald Green, Terence O'Keeffe, Andrew L. Tang, Gary Vercruysse, Peter Rhee

**Affiliations:** Division of Acute Care Surgery, Department of Surgery, University of Arizona, 1501 N. Campbell Ave., Room 5411, PO Box 245603, Tucson, AZ, USA

**Keywords:** Blunt, Diaphragm, Bilateral, Injury

## Abstract

Blunt diaphragmatic rupture (BDR) is uncommon with a reported incidence range of 1%–2%. The true incidence is not known. Bilateral BDR is particularly rare. We presented a case of bilateral BDR and we think that the incidence is under-recognised thanks to an easily missed and difficult to diagnose right sided injury.

## Introduction

Blunt diaphragmatic rupture (BDR) is uncommon. Contemporary series reported an incidence of only 1% to 2% [1], [2], but the reported incidence might be underestimated, given the paucity of published modern series that liberally use computed tomography (CT) and the theoretical assumption that the diagnosis of right-sided injury can be missed and underreported. *Bilateral* BDR is particularly rare. To our knowledge, 5 case reports have been published thus far (Table 1). We herein present a case of bilateral BDR. In light of our patient's presentation, we believe that bilateral BDR may be underreported because of an easily missed (both intraoperatively and postoperatively) right-sided injury.

**Table 1:** Case-reports of bilateral blunt diaphragmatic rupture.

		Age (years)	Sex	Mechanism	Delay in diagnosis (days)
1.	Salah AA et al.^a^	12	Female	Back seat unrestrained MVC^f^	2
2.	Sirbu H et al.^b^	67	Male	MVC	10
3.	Anderson DW et al.^c^	42	Male	Blunt chest trauma	none^g^
4.	Wyffels PL et al.^d^	17	Male	MVC	none^g^
5.	Bryant LR et al.^e^	19	Male	Front passenger MVC	14
6.	Michailidou et al.	16	Male	Back seat unrestrained MVC	17

a Lung India 2011; 28(3): 212–215.

b Hernia 2005; 9(1): 90–92.

c J Trauma 2002; 52: 560–561.

d Am J Surg 1984; 147: 414–417.

e J Trauma 1978; 18(4): 280–282.

f MVC = motor vehicle collision.

g Both diaphragmatic injuries were discovered during the initial operation.

## Case

**Fig. 1:**
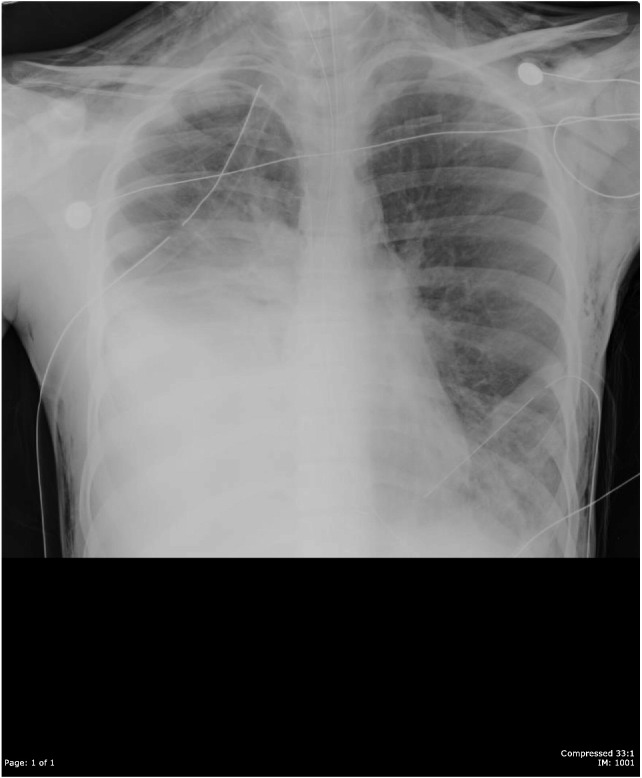
The post-injury daily chest X-ray showed right-sided diaphragmatic abnormality but was nonspecific.

**Fig. 2:**
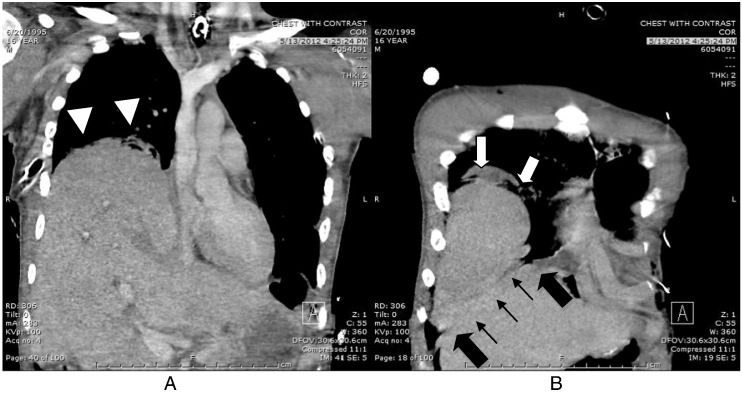
Chest computed tomography demonstrates radiographic signs diagnostic for diaphragmatic injury (Panel A – “Hepatothorax” sign, arrow head. Panel B – “hump” sign, white arrow; “collar” sign, black arrow; “band” sign, black arrow line).

A 16-year-old male unrestrained backseat passenger was brought to our trauma bay after a high-speed motor vehicle collision with a rollover. He suffered multiple injuries, including traumatic brain injury; complex pelvic fractures; spleen, liver, and renal injuries; and left diaphragmatic rupture. His initial chest X-ray (CXR) revealed his stomach herniating inside his left chest. He underwent immediate laparotomy and repair of his left diaphragm. Intraoperatively, the surgeon examined the right diaphragm by palpation and noted no abnormality. Postoperatively, the patient had a prolonged ventilator-dependence. His daily CXR showed right-sided diaphragmatic abnormality (Fig. 1) but was nonspecific. We performed a dynamic ultrasound to evaluate the right diaphragm; the result suggested that the right diaphragm was paralysed, probably secondary to right phrenic nerve injury. On 3 occasions (post-injury day 0, 4, and 15), the patient underwent CT of the chest; none of those 3 scans reported right-sided diaphragmatic injury, despite the presence of “hepatothorax”, as well as “hump”, “band”, and “collar” signs (Fig. 2). Eventually, on post-injury day 17, the patient underwent a diagnostic laparoscopy; we found the liver herniating into the right chest. We reduced the herniated liver and performed an open repair of his right-sided diaphragmatic rupture

## Discussion

Although BDR has been extensively described in the literature over the past several decades [1], [2], little has been written about *bilateral* BDR. Even the diagnosis of *unilateral* BDR remains a challenge for trauma surgeons, particularly when it involves right-sided diaphragmatic injury. Our bilateral BDR case highlights how difficult it is to diagnose a right-sided injury. We would like to underscore the following salient points that the literature has not emphasised.

First, right-sided diaphragmatic injury can be easily missed during exploration and repair of left-sided injury. Of the 5 previous case reports of bilateral BDR, 3 noted the same miss.

Some authors have suggested that right sided injury has not been missed but instead represents a delayed rupture secondary to ischemic changes of devitalised muscles. On the contrary, we believed that our patient's right-sided injury was initially small and escaped our detection during the initial exploration; then, over the course of our patient's hospital stay, his right-sided injury became more prominent and clinically detectable.

Second, diagnosing right-sided BDR is not easy for a number of reasons. With a left-sided injury, the stomach, colon, and spleen can easily herniate into the chest cavity; in contrast, with a right-sided liver, the liver cannot easily herniate into the chest cavity. In fact, the liver is known to have a protective effect, so a small right-sided injury can easily go undetected. Only when the right-sided injury or defect becomes large enough that liver herniates into the chest cavity can a radiologic diagnosis be made.

Another reason that diagnosing the right-sided BDR is not easy is the use of positive-pressure ventilation. Our patient's diagnosis was more difficult, and delayed, because of his prolonged positive-pressure ventilation. Previously, several authors pointed out that positive-pressure ventilation can delay diagnosis [3], [4]. As Karmy-Jones et al. [4] reasoned, only when the thoracic positive pressure comes down (as a patient is being weaned off the ventilator) could the abdominal–thoracic pressure gradient increase and create enough abdominal pressure to push visceral organs into the chest cavity.

Third and finally, we need to stress the utmost importance of trauma surgeons recognising all the CT findings suggestive of possible diaphragmatic injury. Several radiologic studies highlighted some of the key signs, particularly those involving the right side: a continuity defect of the diaphragm; a “dependent viscera” sign which involves visceral organs (bowel, liver) herniating through the diaphragmatic defect and touching the posterior chest wall; a constricting “collar” sign, best seen in a coronal view (Fig. 2, Panel B, black arrow) but sometimes seen in an axial view; a “hump” sign (Fig. 2, Panel B, white arrow), which is specific for a right-sided diaphragmatic injury with herniating liver; and a “band” sign, which is a lucency line or band across the diaphragmatic defect (Fig. 2, panel B, black arrow line). Trauma surgeons must be vigilant concerning these signs: the first or second CT scan may not demonstrate them if the diaphragmatic defect is initially small.

In conclusion, our patient's right-sided injury was missed intraoperatively and remained difficult to diagnose postoperatively, adding to the evidence that bilateral BDR is most likely underreported. Because right-sided injury can so easily go unnoticed, trauma surgeons must be aware of all pertinent CT signs, in order to make an accurate diagnosis early and avoid complications.
